# Are people aware of the link between alcohol and different types of Cancer?

**DOI:** 10.1186/s12889-021-10780-2

**Published:** 2021-04-15

**Authors:** Collin M. Calvert, Traci Toomey, Rhonda Jones-Webb

**Affiliations:** Division of Epidemiology and Community Health, School of Public Health, University of Minnesota, 1300 S. 2nd Street, Suite 300, Minneapolis, MN 55454-1015 USA

## Abstract

**Background:**

Alcohol consumption is causally linked to several different types of cancer, including breast, liver, and colorectal cancer. While prior studies have found low awareness of the overall alcohol-cancer link, few have examined how awareness differs for each type of cancer. Greater awareness of risks associated with alcohol use may be a key factor in reducing alcohol-related cancer incidence.

**Methods:**

We surveyed 1759 people of legal drinking age at the 2019 Minnesota State Fair. We used multivariable generalized linear models and linear regression models with robust standard errors to investigate factors associated with alcohol-cancer risk awareness. Models were fit examining predictors of overall awareness of alcohol as a risk factor for cancer, and prevalence of awareness of alcohol as a risk factor for specific types of cancer.

**Results:**

Prevalence of awareness varied by cancer type, with awareness of alcohol causing liver cancer having the highest prevalence (92%) and awareness of alcohol causing breast cancer having the lowest prevalence (38%). Factors associated with awareness of alcohol-cancer risk differed by type of cancer.

**Conclusions:**

In general, awareness of the risk of alcohol for certain types of cancer was low to moderate, reflecting a need to inform people not only that alcohol increases risk of cancer, but which types of cancer are most highly associated alcohol.

**Supplementary Information:**

The online version contains supplementary material available at 10.1186/s12889-021-10780-2.

## Background

Cancer remains a leading cause of death in the U.S., second only to heart disease [1] The American Cancer Society estimates that in 2020 there will be over 1.8 million new cancer cases and over 600,000 cancer deaths [2]; this represents an increase from 1.6 million new cases [2] and 599,000 deaths [1] in 2017. Cancer incidence varies depending on sociodemographic characteristics and the type of cancer. For example, women are at greater risk for breast cancer than men [3], while men are at higher risk of colorectal cancer [4]. Cancer morbidity and mortality are also disproportionately higher for racial/ethnic minority groups and people with lower incomes [5–7].

Alcohol consumption is causally linked to several types of cancer. An estimated 19,500 of U.S. cancer deaths (3.5%) in 2009 were attributable to alcohol consumption [8]. As of June 2020 the American Cancer Society (ACS) recommends not drinking alcohol to lower cancer risk [9]. ACS specifies seven types of cancer that are linked to alcohol consumption including mouth, throat, laryngeal, esophageal, liver, colorectal, and breast cancer [10].

Many epidemiologic studies have found an association between alcohol use and these different cancer types [11, 12]. For example, a meta-analysis of 112 studies examining alcohol consumption and liver cancer risk found that drinkers had a 17% greater risk of liver cancer compared to people who never drank or drank infrequently [13]. Using data from the Nurses’ Health Study, researchers found that three to six drinks of alcohol per week was associated with a 15% increased risk of breast cancer [14]. In addition, a study of patients with early-stage breast cancer found that consuming three or more drinks of alcohol increased risk of breast cancer recurrence compared to nondrinkers [15]. There may also be a dose-response relationship between alcohol and cancer. Bagnardi et al. examined the relationship between alcohol consumption and 23 cancer types using a meta-analysis, and found increased risk for oral, pharyngeal, esophageal, colorectal, laryngeal, and breast cancers as consumption increased [16]. Cancers associated with increased alcohol consumption are preventable.

Greater awareness of risks associated with alcohol use may be a key factor in reducing alcohol-related cancer incidence. Based on the Transtheoretical Model (or Stages of Change model), awareness of how a behavior is problematic or produces negative health consequences is the first step to someone changing their behavior in a health-positive way [17]. For example, a repeated cross-sectional survey in Australia showed that people who did not perceive alcohol as a risk factor for cancer had greater odds of excess drinking (i.e., more than two drinks per day on average) [18]. Additionally, mass media campaigns designed to increase awareness of the negative consequences of drinking, including cancer, have been shown to be effective in reducing harmful drinking [18–21]. In a recent study by Weerasinghe et al., cancer warning labels applied to alcoholic beverages at a liquor store in Canada increased support for alcohol policies (e.g., minimum unit pricing) among store patrons [19]. On the other hand, a meta-analysis of experimental studies around risk perception and risky behaviors found that heightening risk appraisal alone did not reliably change alcohol consumption [20]. However, the authors note that this may be due to the limited number of studies available for the meta-analysis, and found that risk appraisal was effective when supplemented with heightened self-efficacy for executing recommended behaviors. An alcohol and cancer awareness mass media campaign conducted in Western Australia after this meta-analysis showed a similar increase in knowledge about the cancer risk of alcohol consumption but no reductions in drinking [21].

Awareness of cancer risk from alcohol may relate to their experiences with alcohol-related problems. Using data from the 2010 National Alcohol Survey, Greenfield et al. found that respondents who experienced more harms from second-hand drinking (e.g., involved in a motor vehicle crash with a drunk driver, assaulted or had property vandalized by someone who had been drinking) were more likely to support alcohol policies (e.g., alcohol taxes, beverage warning labels) [22]. These findings suggest that cancer survivors who have been affected by other’s drinking may be more sensitive to the association between alcohol use and risk of cancer. Risk awareness also may relate to propensity for seeking screening and cancer treatment as well as taking prevention measures. In a literature review of risk perception and cancer screening behaviors, Vernon found that women who had a higher awareness of their risk for breast cancer were more likely to undergo mammography screening [23]. If people are aware of the cancer risks associated with alcohol consumption, they may be more likely to reduce their consumption accordingly and engage in other cancer prevention measures.

There are few studies examining alcohol-cancer risk awareness in the U.S. Peacey et al. surveyed female university students across 23 countries from 1999 to 2001, and found that an average of 3.3% were aware that alcohol is a risk factor for breast cancer [24]. Among the sample of U.S. students in this study, awareness was higher – but only averaged at approximately 10%, and the sample was not representative of the general U.S. population. Another study surveyed participants from the New York City metropolitan area visiting four east coast dental schools for oral cancer screening, and found that only 25% were aware that alcohol was a risk factor for oral cancer [25]. Similar studies conducted in other countries have found similarly low prevalence of awareness of the link between alcohol and cancer. A study of oral cancer awareness, this time among undergraduate medical and dental students in Scotland, found that while 94% of dental students were aware of alcohol as a risk factor for oral cancer, only 33% of medical students were aware of this connection [26]. A report by Cancer Care Ontario showed that two-thirds of Canadians were unaware of the cancer risk from alcohol consumption [27]. Data for this report came from the Canadian Community Health Survey, considered representative of 98% of the Canadian population aged 12 years and older. Several other studies conducted outside of the U.S. (Sri Lanka, the UK) also found low awareness of the connection between alcohol and some types of cancer [28–31]. Using a representative sample of 2100 English adults, Buykx et al. found that awareness varied depending on the type of cancer: 80% believed that alcohol can cause liver cancer, but only 18% believed it could cause breast cancer [29]. More research is needed to understand the prevalence of awareness for the alcohol-cancer link, as well as heterogeneity in awareness across demographic groups and types of cancer.

The objectives of this study were to: 1) assess prevalence of alcohol-cancer risk awareness, 2) determine whether alcohol-cancer risk awareness varies by cancer type, and 3) identify differences in alcohol-cancer risk awareness by different demographic variables (e.g., race/ethnicity, gender), risk behaviors (i.e., alcohol and tobacco use), and experiences with cancer. We hypothesized that overall alcohol-cancer risk awareness will be low, but will vary by the type of cancer (e.g., awareness for liver cancer will be higher than for breast cancer). We also hypothesize that relationships between participant characteristics and alcohol cancer awareness will also vary by the type of cancer given cancer risk varies by demographics (e.g., women will be more likely to know that alcohol causes breast cancer since they are at higher risk).

## Methods

### Data collection and measures

We collected data at the Minnesota State Fair Driven to Discover (D2D) Research Facility over the course of five days (in August and September of 2019). We selected the Minnesota State Fair because it attracts large numbers of Minnesotans from across the state and has a research facility specifically designed for conducting large-scale surveys. In 2019, a record 2,126,551 people attended the fair [32]. Over 27,700 fairgoers participated in studies within the D2D Facility [33].

Volunteer staff recruited participants at the D2D Facility, explained the survey procedures and participation incentives. Interested fairgoers were then screened for eligibility; participants were required to be of legal drinking age (21+ years) and be able to read English fluently. Fairgoers who were eligible then reviewed consent information prior to answering survey questions. After completing their survey, participants were given a University of Minnesota tote bag for participating in the study. Participants were not informed prior to beginning the survey that the study was designed to measure their knowledge regarding alcohol as a risk factor for cancer. Instead, participants were told that the purpose of the study was to measure cancer risk knowledge more broadly. This was done so that participants were not biased towards identifying alcohol as a risk factor.

The survey (see Additional File 3) was designed to take approximately 5–10 min to complete. Questions were developed based on prior surveys measuring alcohol-cancer risk awareness [29]. Additionally, we adopted questions from the Behavioral Risk Factor Surveillance System (BRFSS) survey [34] to measure substance use and demographic characteristics. Participants entered responses on Apple iPads, which were automatically recorded in REDCap, a secure web-based application for managing survey data and databases. All study procedures were approved by the University of Minnesota Institutional Review Board.

### Measures

#### Dependent variables

Two types of dependent variables were analyzed: 1) awareness of alcohol as a risk factor for cancer; and 2) awareness of the association between alcohol use and different types of cancers. For the former, participants were asked to identify risk factors (including alcohol) for cancer among a list of seven behaviors and conditions that either do or do not increase cancer risk (i.e., smoking cigarettes, stress, exposure to sunlight/ultraviolet radiation, eating genetically modified foods, obesity, drinking coffee). Participants could indicate, “Yes,” “No,” or “Don’t know.” For this measure, we were interested in assessing whether participants were aware that alcohol is a risk factor for cancer in comparison to their knowledge about what other behaviors/conditions are associated with increased risk of cancer. For the second type of dependent measure, participants were also asked to specifically identify which types of cancer were associated with alcohol consumption (stomach, ovarian, breast, mouth and throat, brain, colon and rectal, laryngeal, esophageal, liver, bladder) using the same three answer options as the prior question. Only some of these options (i.e., breast, mouth and throat, colon and rectal, laryngeal, esophageal, liver) are associated with alcohol. These variables were dichotomized such that “Yes” answers meant a participant was aware, and “No” or “Don’t know” answers meant a participant was not aware. In each case, correct responses were coded 1, while incorrect and don’t know responses were coded 0.

#### Independent variables

In analyses examining factors associated with alcohol-cancer risk awareness, independent variables included substance use behaviors, cancer history, and demographic characteristics. We specifically asked questions about alcohol consumption and tobacco use. For alcohol, we measured frequency of consumption and average daily amount consumed in the past 30 days, which were combined into a quantity-frequency measure by multiplying the two variables together. We also measured frequency of alcohol binge drinking in a 30-day period. Due to skewness, we dichotomized this to identify people who binge drank versus people who did not binge drink. Tobacco use frequency was measured using a single item that asked about frequency tobacco product use (“Do you currently use a tobacco product (cigarettes, chewing tobacco, snuff, snus) every day, some days, or not at all?”) and had four answer options (“Every day,” “Some days,” “Not at all,” “Don’t know”). Two questions were about cancer history: “Have you ever been or are you currently diagnosed with cancer? (Yes/No/Prefer not to answer)” and “That you know of, has anyone in your family been diagnosed with cancer? (Yes/No/Don’t know/Prefer not to answer).” Finally, we asked several questions about participant demographics (educational attainment [< 9 years – high school diploma/GED; Some college – Associate’s degree; Bachelor’s degree; Graduate degree], age [in 10-year intervals], ethnicity [Hispanic/Latino; non-Hispanic/Latino], gender [men/women], and race [White; African American; American Indian; Asian; Not White, Black, American Indian, or Asian]).

### Statistical analysis

Descriptive statistics were used to determine the prevalence of alcohol-cancer risk awareness, such as the proportion of “Yes” responses for each risk awareness question. For binary dependent variables, we measured the probability of knowing that alcohol is a risk factor for cancer using multivariable generalized linear models (GLM) with an identity link and binomial distribution. When GLM models did not converge, we fit linear regression models with robust standard errors instead. For continuous dependent variables (i.e., index score), we used multivariable linear regression models. Independent variables included a quantity-frequency measure of alcohol consumed the past 30 days, whether or not someone binge drank the past 30 days, frequency of tobacco use, if a participant has ever been diagnosed with cancer, whether a family member has ever been diagnosed with cancer, and demographic variables.

Our primary analyses included the full sample of participants. However, we also conducted a sensitivity analysis to determine whether awareness differed between survey straight liners (participants who select the same response for an entire set of survey items) and non-straight liners. Seven percent (*n* = 135) of the full sample were identified as straight liners. Because straight-lining can indicate a lack of effort or engagement on the part of a participant when answering questions [35], we wanted to ensure measurement of awareness was internally valid. Behavioral and demographic characteristics of straight liners were compared to characteristics of non-straight liners using t-tests (for continuous variables) and Chi-square tests (for categorical variables). Models for overall alcohol and cancer awareness were re-fit with only non-straight liners and results were compared to models that incorporated the full sample of participants. Participants with missing data for variables included in regression models were excluded from analyses.

All analyses were conducted using Stata version 16.

## Results

A total of 1759 fairgoers completed our survey. Six of the participants were excluded because their reported age was below the screening threshold of 21+ years, resulting in 1753 total observations for analyses.

### Sample characteristics

The median age of participants was 52 years. Most participants identified as white (87%) and not Hispanic or Latino (96%) (Table 1). Over half of participants had attained a bachelor’s degree or higher (66%). The majority of participants identified as women (65%). Nearly all participants (95%) had consumed alcohol at some point in their lives (Table 1). In the past 30 days, participants reported a median of four drinking days (i.e., days in which they consumed some amount of alcohol) and, on those days, one drink was most typical. Nearly one-third (32%) of participants reported binge drinking alcohol in the past 30 days. Ninety-two percent of participants reported no tobacco use (*n* = 1578) while 4% reported every day tobacco use. Overall, 12% of participants said they had been diagnosed with cancer at some point in their lives while 79% said they knew a family member who had been diagnosed with cancer.

**Table 1:** Sample characteristics

Percentages (%) and Median (Interquartile range)	
Drinkers (ever in lifetime)	95%
Days drank (in the last 30)	4 (9)
Drinks per day (in the last 30)	1 (1)
Binge drinker	32%
Frequency of using a tobacco product	
Every day	4%
Some days	4%
Not at all	92%
Don’t know	1%
Ever been diagnosed with cancer?	12%
Ever had a family member diagnosed?	79%
Education	
Less than 9th-High school/GED	7%
Some college-Associate’s	27%
Bachelor’s	36%
Graduate	30%
Hispanic/Latine	4%
Race	
White	87%
Black	2%
American Indian	1%
Asian	6%
Not White, Black, American Indian, or Asian	4%
Age	52 (27)
Gender	
Woman	65%
Man	35%
Alcohol is risk factor for cancer (answered “Yes”)	87%
Cancer types (answered “Yes”)	
Breast cancer is associated with alcohol	38%
Mouth/throat cancer is associated with alcohol	66%
Colon/rectal cancer is associated with alcohol	66%
Laryngeal cancer is associated with alcohol	56%
Esophageal cancer is associated with alcohol	63%
Liver cancer is associated with alcohol	92%

Table 2 shows several demographic characteristics and drinking patterns for the state of Minnesota and for this study sample. Compared to the Minnesota population, study participants were more likely to identify as white, women, older, more highly educated and to have consumed alcohol at some time in their lives when compared to the population of Minnesota.

**Table 2:** Sample and Minnesota drinking patterns and demographics

	Study sample	Minnesota
Drinkers (ever in lifetime)	95%	61%
Binge drinker	32%	20%
Education		
Less than 9th-High school/GED	7%	28%
Some college-Associate’s	27%	35%
Bachelor’s degree or higher	36%	25%
Graduate	30%	12%
Hispanic/Latine	4%	6%
Race		
White	87%	84%
Black	2%	7%
American Indian	1%	1%
Asian	6%	5%
Not White, Black, American Indian, or Asian	4%	3%
Age 65+	21%	16%
Gender		
Woman	65%	50%
Man	35%	50%

^1^Data retrieved from the MN Department of Health webpage, “Alcohol and Other Drugs Quick Facts” at URL: https://www.health.state.mn.us/communities/alcohol/data/quickfacts.html

^2^Data retrieved from the MN Office of Higher Education webpage, “Educational Attainment Data” at URL: https://www.ohe.state.mn.us/sPages/educ_attain.cfm#:~:text=Minnesota%20ranks%202nd%20(50%20%,an%20associate%20degree%20or%20higher

^3^Data retrieved from the MN Department of Health webpage, “Alcohol and Other Drugs Quick Facts” at URL: https://www.census.gov/quickfacts/MN

### Prevalence of alcohol Cancer awareness

Overall, 87% of participants in the full sample were aware that alcohol consumption is a risk factor for cancer (Table 1). Alcohol-cancer risk awareness varied by cancer type. The highest alcohol-cancer risk awareness was for liver cancer (92%) while breast cancer had the lowest awareness (38%). Awareness for other cancer types ranged from 51% (Laryngeal) to 66% (colon/rectal and mouth/throat).

### Predictors of alcohol-Cancer risk awareness

Overall alcohol cancer risk awareness was less common among men and African American participants. For example, men had a 5% lesser probability of alcohol-cancer risk awareness compared to women (mean: − 0.05; confidence interval [CI]: − 0.09, − 0.01) (Table 3); and African American participants, had a 24% lesser probability of awareness compared to participants from other racial/ethnic groups (mean: -0.24; CI: − 0.46, − 0.03).

**Table 3:** Factors Associated with Knowledge of Alcohol-Cancer Risk

	Correctly identify alcohol as risk factor for cancer	
	Full sample	Straight liners excluded
	Adjusted difference in mean probability (95% CI)	
**Substance use**		
Quantity-frequency of drinking	0.00 (− 0.00, 0.00)	0.00 (− 0.00, 0.00)
Binge drinker	0.00 (− 0.04, 0.04)	0.00 (− 0.04, 0.04)
Not a binge drinker	Ref	Ref
Frequency of tobacco use = Every day	−0.03 (− 0.12, 0.07)	− 0.01 (− 0.11, 0.09)
Frequency of tobacco use = Some days	−0.01 (− 0.12, 0.09)	−0.02 (− 0.14, 0.09)
Frequency of tobacco use = Not at all	Ref	Ref
**Cancer history**		
History of cancer	0.02 (−0.04, 0.07)	0.02 (− 0.04, 0.07)
No known history of cancer	Ref	Ref
Family history of cancer	0.02 (−0.02, 0.05)	0.02 (− 0.02, 0.06)
No known family history of cancer	Ref	Ref
**Demographic and socioeconomic characteristics**		
Educational attainment = Some college-Associate’s	0.03 (− 0.05, 0.11)	0.04 (− 0.05, 0.13)
Educational attainment = Bachelor’s	0.06 (− 0.02, 0.14)	0.07 (− 0.02, 0.16)
Educational attainment = Graduate	0.06 (− 0.02, 0.14)	0.08 (− 0.01, 0.17)
Educational attainment = High school diploma/GED	Ref	Ref
Age (intervals of 10 years)	− 0.00 (− 0.02, 0.01)	0.00 (− 0.02, 0.01)
Gender = Man	**− 0.05 (− 0.09, − 0.01)**	**− 0.05 (− 0.09, − 0.01)**
Gender = Woman	Ref	Ref
Hispanic/Latine	− 0.02 (− 0.12, 0.08)	− 0.01 (− 0.11, 0.09)
Not Hispanic/Latine	Ref	Ref
Race = Black	**− 0.24 (− 0.46, − 0.03)**	**− 0.26 (− 0.47, − 0.04)**
Race = American Indian	− 0.09 (− 0.32, 0.15)	−0.16 (− 0.46, 0.14)
Race = Asian	−0.03 (− 0.11, 0.05)	−0.03 (− 0.12, 0.06)
Race = Not White, Black, Native, or Asian	0.04 (− 0.05, 0.12)	0.03 (− 0.06, 0.12)
Race = White	Ref	Ref

**Bold** = *P* < 0.05

Alcohol-cancer risk awareness and cancer type. Table 4. shows results from models predicting alcohol-cancer risk awareness by cancer type in the full sample of participants. Several relationships emerged across different cancer types. Participants who binge drank in the past 30 days were more likely to identify breast cancer as a risk of alcohol consumption (mean: 0.07; CI: 0.01, 0.14), but less likely to identify mouth and throat cancers as associated with alcohol use (mean: -0.09; CI: − 0.16, − 0.02). Having been diagnosed with cancer increased awareness only for esophageal cancer while knowing a family member with a cancer history increased awareness for mouth and throat and esophageal. Identifying as a man was associated with decreased likelihood of awareness for breast (mean: -0.14; CI: − 0.19, − 0.08) and laryngeal cancer (mean: -0.06; CI: − 0.11, − 0.00). American Indian participants were more likely to be aware of liver cancer risk (mean: 0.07; CI: 0.03, 0.10).

**Table 4:** Factors Associated with Knowledge of Alcohol-Cancer Risk by Cancer Type (Full Sample)

	Breast cancer	Mouth/throat cancer	Colon/rectal cancer	Laryngeal cancer	Esophageal cancer	Liver cancer	Index score
Quantity-frequency of drinking	0.00 (−0.00, 0.00)	0.00 (− 0.00, 0.00)	0.00 (− 0.00, 0.00)	0.00 (− 0.00, 0.00)	−0.00 (− 0.00, 0.00)	−0.00 (− 0.00, 0.00)	0.00 (− 0.00, 0.01)
Binge drinker	**0.07 (0.01, 0.14)**	**− 0.09 (− 0.16, − 0.02)**	−0.00 (− 0.07, 0.06)	0.03 (− 0.05, 0.10)	−0.01 (− 0.08, 0.06)	−0.02 (− 0.06, 0.01)	−0.07 (− 0.37, 0.23)
Not a binge drinker	Ref	Ref	Ref	Ref	Ref	Ref	Ref
Frequency of tobacco use							
Every day	−0.09 (−0.21, 0.03)	−0.03 (− 0.16, 0.10)	0.02 (− 0.11, 0.15)	−0.02 (− 0.16, 0.11)	−0.08 (− 0.22, 0.06)	0.02 (− 0.05, 0.08)	−0.05 (− 0.65, 0.54)
Some days	0.09 (−0.06, 0.23)	−0.01 (− 0.15, 0.14)	0.06 (− 0.08, 0.19)	0.09 (− 0.06, 0.24)	0.05 (− 0.09, 0.20)	0.01 (− 0.08, 0.09)	0.47 (− 0.12, 1.05)
Not at all	Ref	Ref	Ref	Ref	Ref	Ref	Ref
History of cancer	0.02 (−0.06, 0.09)	0.06 (−0.02, 0.13)	0.03 (− 0.05, 0.10)	0.06 (− 0.02, 0.14)	**0.09 (0.02, 0.16)**	0.02 (− 0.02, 0.05)	0.32 (− 0.03, 0.68)
No known history	Ref	Ref	Ref	Ref	Ref	Ref	Ref
Family history of cancer	0.02 (−0.03, 0.07)	**0.06 (0.00, 0.11)**	0.02 (−0.04, 0.07)	0.02 (− 0.03, 0.08)	**0.06 (0.01, 0.12)**	0.02 (− 0.01, 0.05)	0.20 (− 0.04, 0.44)
No known family history	Ref	Ref	Ref	Ref	Ref	Ref	Ref
Educational attainment							
Some college-Associate’s	−0.06 (−0.18, 0.05)	−0.06 (− 0.16, 0.05)	0.06 (− 0.06, 0.17)	0.01 (− 0.10, 0.12)	0.07 (− 0.04, 0.19)	0.00 (− 0.06, 0.06)	−0.07 (− 0.56, 0.42)
Bachelor’s	−0.09 (− 0.20, 0.02)	−0.10 (− 0.20, 0.01)	0.06 (− 0.05, 0.17)	0.06 (− 0.05, 0.17)	0.04 (− 0.07, 0.15)	−0.00 (− 0.06, 0.06)	0.09 (− 0.39, 0.58)
Graduate	−0.01 (− 0.13, 0.10)	−0.09 (− 0.20, 0.01)	0.05 (− 0.06, 0.16)	0.03 (− 0.08, 0.15)	0.04 (− 0.08, 0.15)	0.01 (− 0.05, 0.07)	0.09 (− 0.40, 0.58)
High school diploma/GED	Ref	Ref	Ref	Ref	Ref	Ref	Ref
Age (intervals of 10 years)	**0.06 (0.04, 0.07)**	−0.00 (−0.02, 0.01)	0.01 (−0.01, 0.03)	**0.04 (0.02, 0.06)**	0.01 (−0.00, 0.03)	− 0.01 (− 0.02, 0.00)	0.06 (− 0.02, 0.13)
Hispanic/Latine	0.04 (−0.11, 0.19)	−0.03 (− 0.18, 0.11)	−0.07 (− 0.21, 0.08)	−0.02 (− 0.17, 0.14)	−0.04 (− 0.19, 0.11)	−0.03 (− 0.10, 0.05)	−0.02 (− 0.64, 0.60)
Not Hispanic/Latine	Ref	Ref	Ref	Ref	Ref	Ref	Ref
Gender							
Man	**−0.14 (−0.19, − 0.08)**	0.00 (− 0.05, 0.06)	0.04 (− 0.01, 0.10)	**−0.06 (− 0.11, − 0.00)**	−0.01 (− 0.06, 0.05)	−0.00 (− 0.03, 0.03)	−0.13 (− 0.36, 0.10)
Woman	Ref	Ref	Ref	Ref	Ref	Ref	Ref
Race							
Black	−0.13 (−0.30, 0.05)	−0.01 (− 0.21, 0.19)	0.01 (− 0.19, 0.21)	−0.05 (− 0.26, 0.17)	−0.15 (− 0.37, 0.07)	−0.07 (− 0.22, 0.08)	0.69 (− 0.08, 1.47)
American Indian	0.24 (−0.01, 0.48)	0.08 (−0.19, 0.34)	0.09 (− 0.16, 0.35)	0.04 (− 0.23, 0.31)	0.06 (− 0.21, 0.32)	**0.07 (0.03, 0.10)**	0.82 (− 0.22, 1.86)
Asian	0.04 (−0.08, 0.16)	−0.11 (− 0.23, 0.01)	−0.04 (− 0.16, 0.09)	−0.09 (− 0.21, 0.03)	−0.12 (− 0.24, 0.01)	−0.04 (− 0.11, 0.03)	−0.42 (− 0.93, 0.10)
Not White, Black, Indian, or Asian	0.01 (−0.13, 0.15)	0.08 (−0.04, 0.21)	0.05 (− 0.08, 0.18)	0.06 (− 0.08, 0.20)	0.03 (− 0.10, 0.17)	−0.00 (− 0.07, 0.06)	0.21 (− 0.38, 0.80)
White	Ref	Ref	Ref	Ref	Ref	Ref	Ref

**Bold** = *P* < 0.05

### Sensitivity analysis

There were some differences between straight liners and non-straight liners regarding alcohol consumption (see Additional File 1). Straight liners tended to drink more, on average, than non-straight liners and were more likely to binge drink. Across all other behaviors and demographic characteristics, there were no significant differences. In models examining awareness of alcohol as a risk factor for specific cancer types, a small portion of estimates either lost or gained significance (see Additional File 2). Binge drinking was no longer significantly associated with increased awareness that alcohol is a risk factor for breast cancer, and identifying as a man became non-significant for decreased awareness that alcohol is a risk factor for laryngeal cancer. Conversely, Asian participants had lower awareness of mouth and throat, laryngeal, and esophageal cancer risk. Despite changes in significance, the direction of all estimates remained consistent and magnitude changed only marginally.

## Discussion

Previous studies of alcohol and cancer awareness have been limited to only a select few of the cancer types associated with alcohol. This study builds on prior research of awareness that alcohol consumption confers risk of cancer by examining all types of cancer linked to alcohol. Our findings partially support our hypotheses: prevalence of awareness of the association between alcohol use and cancer varied by type of cancer, as did the behavioral and demographic characteristics that predicted alcohol-cancer risk awareness. However, while we hypothesized that overall awareness that alcohol is associated with cancer would be low, the prevalence of awareness in our sample was high.

In our sample, 87% of participants correctly identified alcohol consumption as a risk factor for cancer. This finding is inconsistent with prior studies, which have shown low awareness of this risk (< 50%) [29, 36]. There are several possible explanations for the inconsistency in results between our study and prior studies. First, our sample was highly educated, with the majority of participants having a Bachelor’s (36%) or graduate degree (30%). Second, participants were primed to consider cancer and cancer risk factors by the title of the study displayed at the fair: “What do you know about risks for cancer?” Consent forms also informed participants that the study was regarding cancer risks. The result may have been that people who chose to participate were more knowledgeable about cancer, and those less knowledgeable decided not to participate. Third, participants were prompted with a pre-determined set of possible risk factors rather than being asked open-ended questions. Prompted questions about cancer awareness tended to show higher awareness than un-prompted, open-ended questions in previous research [36]. Future studies of awareness of alcohol and cancer risk would benefit from using both prompted and un-prompted questions to gain a more in-depth understanding of knowledge around alcohol-cancer risk.

There was some consistency between our findings and prior studies when examining awareness across different types of cancer. For example, breast cancer awareness in our sample was low (38%), and awareness of liver cancer was the only cancer type with prevalence greater than 66%. Similar to our results, Buykx et al. measured awareness across several types of cancer and found breast cancer to be the lowest (18%), followed by colorectal (39%), mouth and throat (48%), and liver cancer (80%) among 2100 adults in England [29]. High awareness of the connection between alcohol and liver cancer may relate to awareness that alcohol causes liver problems, such as cirrhosis of the liver. In Buykx et al., 95% of participants believed that alcohol could cause liver disease. In addition, people may surmise that alcohol can cause damage to the mouth and throat given their direct contact with alcohol when consuming. Awareness for breast cancer may also be low because of this and other factors. Alcohol corporations advertising their products using breast cancer awareness imagery and linking donations with product sales may undermine efforts to raise awareness about the link between alcohol and breast cancer [37].

African American participants were less likely to identify alcohol as a risk factor for cancer. Structurally racist barriers for African Americans [38], including mistrust of the healthcare system, a lack of access to healthcare [39] and poor quality or availability of cancer screening [7, 40], may explain this finding. However, it is important to note that estimates were also imprecise due to the small number of participants who identified as African American. In addition, there were no significant differences in alcohol-cancer awareness between African American and white participants across different cancer types. Because sample sizes in each racial/ethnic group (besides White) were low, estimates comparing across groups may be unreliable.

Binge drinking was significantly associated with increased probability of awareness that alcohol and cancer are linked, but decreased awareness for mouth and throat cancers (associations for all other cancer types were non-significant). Few studies have examined the relationship between alcohol consumption and awareness of cancer risk from alcohol. In a sample of women aged 15–44 years (*n* = 10,940) in the U.S., current drinkers were less likely to be aware that alcohol causes breast cancer compared to non-drinkers (27% vs. 21%) [41]. Likelihood of awareness of the link between alcohol and breast cancer risk between non-drinkers and binge-drinkers, however, was more similar (27% vs. 25%). How drinking behaviors predict awareness of cancer risk, and whether this varies by the type of cancer, is yet unknown. More research is needed to examine this question across different populations.

### Limitations

These findings should be considered in light of several limitations. Participants were not randomly selected; rather, we used a convenience sample of fair attendees. Participant self-selected to come to the D2D building and to participate in this specific study. Thus, results may not be generalizable to a broader population. Among all participants, 66% had a Bachelor’s or Graduate degree, and 87% were white. In Minnesota in 2018, only 35% of people had a Bachelor’s or Graduate degree, and 80% identified as white [42]. Because these factors are associated with cancer risk awareness, our estimates for the proportion of people aware that alcohol is a risk factor for cancer may overestimate awareness compared to the general population. In addition, mention of our study being focused on cancer may have primed participants to select ‘Yes’ for each risk factor (regardless of whether or not they truly knew alcohol is a risk factor). To address this, we conducted our analyses on both the full sample of participants and to a subsample that excluded straight liners. While we did assess how race/ethnicity is associated with alcohol-cancer awareness, this measure does not fully capture the effects of structural racism (i.e., the interplay of policies, institutions, and cultural norms that perpetuates racial group inequality). In analyses, race/ethnicity may be considered an imperfect proxy for the social and environmental forces that disproportionately affect health care and other resources available to indigenous and people of color [43]. Additionally, given the limited number of participants across different racial/ethnic groups, we were unable to further explore the basis of any race/ethnic associated differences. Future studies should analyze the role of race/ethnicity and structural racism as a predictor or alcohol-cancer risk awareness, preferably with a larger multi-ethnic sample of participants.

Given that some participants may have answered “Yes” or “No” to all survey items rather than attending to each question, we conducted a sensitivity analysis by excluding straight liners (*n* = 135). However, models which exclude straight liners may be prone to collider stratification bias (a type of selection bias) [44]. Being considered a straight liner is related to our outcome variables (awareness), and – as shown in Additional File 1 – also associated with a number of behavioral and demographic characteristics. When we excluded straight liners for our sensitivity analysis (effectively conditioning on a collider), we may have introduced bias in our estimates. This may explain why significance changed for some estimates between the full sample and the sensitivity analysis (particularly those that were not significant in the full sample but became significant in the sensitivity analysis). Because choosing either option represents a trade-off without a clear best choice, we elected to provide results from both the full sample and the sample with straight liners excluded. Overall, results did not change drastically between the full sample and subsample excluding straight liners.

## Conclusions and implications

Our results indicate that awareness of the link between alcohol consumption and cancer varies greatly by the type of cancer. Surprisingly, we found high awareness that alcohol was a cancer risk factor (though this may not reflect true awareness, but rather priming). However, awareness for certain types of cancer was low to moderate, reflecting a need to inform people not only that alcohol increases risk of cancer, but which types of cancer alcohol increases the risk of. Part of the reason for this low awareness may be the connections between alcohol corporations and cancer charities. Martin and Giesbrecht document how several alcohol corporations have linked their brand to cancer charity organizations through marketing materials (e.g., pink ribbons on beverage labels) and donations to cancer charities based on product sales [37]. This sends a mixed message to consumers concerned about cancer risk. For example, the authors note that cancer charities accepting funding from alcohol corporations, particularly when funds are linked to volume of alcohol purchased may undermine messaging that alcohol is a cause of cancer. Cancer charities and other major organizations involved in cancer awareness campaigns should adopt guidelines that discourage alcohol sponsorships.

Screening for alcohol use during healthcare visits and informing patients of this risk may be one way to increase awareness. In addition, increased awareness of the alcohol-cancer link may help generate support for alcohol policies that may be key to reducing alcohol consumption and future alcohol-related problems [19, 45]. When made aware that alcohol causes cancer, support increased for minimum unit pricing per standard drink of alcohol. This and similar policies that affect beverage pricing (e.g., taxes) have been shown to reduce alcohol consumption and sales, and ultimately reduce alcohol-related health problems [46]. However, more information is needed to identify subgroups who may benefit most from awareness campaigns or other interventions to reduce consumption and alcohol-related cancers.

## Supplementary Information

**Additional File 1: Supplementary Table 1.** Bivariate analysis for straight-liners and non straight-liners. A table comparing straight liners and non-straight liners across all covariates. Percentages, means, and the results of bivariate hypothesis tests are included.

**Additional file 2: Supplementary Table 2.** Factors Associated with Knowledge of Alcohol-Cancer Risk by Cancer Type (Straight Liners Excluded). A table formatting the same way as Table 3 in the manuscript, but excluding straight liners. Mean differences and confidence intervals are included.

**Additional file 3.** Survey Questionnaire. The questionnaire developed by research staff. Questions were adapted from the Behavioral Risk Factor Surveillance System and prior studies of alcohol-cancer awareness. Citations for these are included in the manuscript text.

## Data Availability

The datasets generated and/or analyzed during the current study are not publicly available to protect the privacy of participants, but are available from the corresponding author on reasonable request.

## Abbreviations

ACS: American Cancer Society
D2D: Driven to Discover
BRFSS: Behavioral Risk Factor Surveillance System
GLM: Generalized linear model
CI: Confidence interval
