# Supplementary material for: Are people aware of the link between alcohol and different types of Cancer?
Source: BMC Public Health. 2021 Apr 15;21:734. doi: 10.1186/s12889-021-10780-2 (PMC8051079; doi:10.1186/s12889-021-10780-2)
Supplement: Supplementary file 1 — Additional File 1: Supplementary Table 1. Bivariate analysis for straight-liners and non straight-liners. A table comparing straight liners and non-straight liners across all covariates. Percentages, means, and the results of bivariate hypothesis tests are included. [file 12889_2021_10780_MOESM1_ESM.docx]

**Supplementary Table 1** Bivariate analysis for straight-liners and non straight-liners

Values shown are percentages and means.

|  | Non straight-liners (n=1,619) | Straight liners (n=135) | P-value* |
| --- | --- | --- | --- |
| Abstainer | 4% | 7% | 0.24 |
| # of days drank | 6.46 | 5.76 | 0.32 |
| # of drinks per day | 1.71 | 2.76 | <0.01 |
| Binge drinker | 31% | 40% | 0.04 |
| Tobacco use frequency |  |  | 0.06 |
| Every day | 4% | 3% |  |
| Some days | 4% | 4% |  |
| Not at all | 92% | 90% |  |
| History of cancer | 12% | 12% | 0.80 |
| Family history of cancer | 79% | 76% | 0.49 |
| Education level |  |  | 0.05 |
| <9 years-High school/GED | 8% | 13% |  |
| Some college-Associate’s | 26% | 31% |  |
| Bachelor’s degree | 36% | 35% |  |
| Graduate degree | 30% | 22% |  |
| Hispanic/Latine | 4% | 2% | 0.39 |
| Race |  |  | 0.49 |
| White | 87% | 83% |  |
| Black | 2% | 2% |  |
| American Indian | 1% | 2% |  |
| Asian | 6% | 7% |  |
| None of the above | 4% | 5% |  |
| Age | 49.73 | 50.53 | 0.59 |
| Gender |  |  | 0.28 |
| Man | 93% | 7% |  |
| Woman | 92% | 8% |  |

*Tests generated from chi-square tests for categorical measures, and two-sample t-tests for continuous measures.
