# Supplementary material for: Are people aware of the link between alcohol and different types of Cancer?
Source: BMC Public Health. 2021 Apr 15;21:734. doi: 10.1186/s12889-021-10780-2 (PMC8051079; doi:10.1186/s12889-021-10780-2)
Supplement: Supplementary file 2 — Additional file 2: Supplementary Table 2. Factors Associated with Knowledge of Alcohol-Cancer Risk by Cancer Type (Straight Liners Excluded). A table formatting the same way as Table 3 in the manuscript, but excluding straight liners. Mean differences and confidence intervals are included. [file 12889_2021_10780_MOESM2_ESM.rtf]

Supplementary Table 2  Factors Associated with Knowledge of Alcohol-Cancer Risk by Cancer Type (Straight Liners Excluded)
	Breast cancer 	Mouth/throat cancer	Colon/rectal cancer	Laryngeal cancer	Esophageal cancer	Liver cancer	Index score	
Quantity-frequency of drinking	0.00
(-0.00, 0.00)	0.00
(-0.00, 0.00)	0.00
(-0.00, 0.00)	0.00
(-0.00, 0.00)	0.00
(-0.00, 0.00)	-0.00
(-0.00, 0.00)	0.00
(-0.00, 0.01)	
Binge drinker	0.05
(-0.02, 0.12)	-0.11
(-0.19, -0.04)	-0.02
(-0.10, 0.05)	0.00
(-0.07, 0.08)	-0.03
(-0.11, 0.04)	-0.03
(-0.07, 0.01)	-0.17
(-0.48, 0.15)	
Not a binge drinker	Ref	Ref	Ref	Ref	Ref	Ref	Ref	
Frequency of tobacco use								
Every day	-0.06
(-0.18, 0.06)	-0.01
(-0.15, 0.12)	0.04
(-0.09, 0.18)	0.01
(-0.13, 0.15)	-0.06
(-0.20, 0.08)	0.02
(-0.05, 0.09)	0.05
(-0.56, 0.67)	
								
Some days	0.06
(-0.09, 0.21)	-0.03
(-0.18, 0.13)	0.05
(-0.10, 0.20)	0.07
(-0.09, 0.23)	0.04
(-0.12, 0.20)	-0.00
(-0.09, 0.09)	0.42
(-0.21, 1.05)	
								
Not at all	Ref	Ref	Ref	Ref	Ref	Ref	Ref	
History of cancer	0.02
(-0.05, 0.10)	0.06
(-0.01, 0.14)	0.03
(-0.04, 0.11)	0.07
(-0.02, 0.15)	0.10
(0.02, 0.18)	0.02
(-0.02, 0.06)	0.37
(-0.00, 0.74)	
								
No known history	Ref	Ref	Ref	Ref	Ref	Ref	Ref	
Family history of cancer	0.04
(-0.01, 0.09)	0.07
(0.01, 0.13)	0.02
(-0.04, 0.08)	0.04
(-0.02, 0.10)	0.08
(0.02, 0.13)	0.02
(-0.01, 0.06)	0.24
(-0.01, 0.49)	
								
No known family history	Ref	Ref	Ref	Ref	Ref	Ref	Ref	
Educational attainment								
Some college-Associate's	-0.06
(-0.17, 0.06)	-0.05
(-0.17, 0.06)	0.08
(-0.05, 0.20)	0.03
(-0.09, 0.15)	0.09
(-0.02, 0.21)	0.01
(-0.06, 0.07)	-0.02
(-0.53, 0.50)	
								
Bachelor's	-0.06
(-0.17, 0.05)	-0.09
(-0.20, 0.03)	0.09
(-0.03, 0.21)	0.10
(-0.02, 0.21)	0.07
(-0.05, 0.19)	0.00
(-0.06, 0.07)	0.22
(-0.29, 0.73)	
								
Graduate	0.03
(-0.09, 0.14)	-0.08
(-0.19, 0.04)	0.09
(-0.03, 0.21)	0.08
(-0.04, 0.20)	0.07
(-0.05, 0.19)	0.02
(-0.05, 0.09)	0.26
(-0.26, 0.77)	
								
High school diploma/GED	Ref	Ref	Ref	Ref	Ref	Ref	Ref	
Age (intervals of 10 years)	0.06
(0.04, 0.08)	-0.00
(-0.02, 0.01)	0.01
(-0.01, 0.03)	0.04
(0.02, 0.06)	0.01
(-0.00, 0.03)	-0.01
(-0.02, 0.00)	0.06
(-0.02, 0.14)	
								
Hispanic/Latine	0.11
(-0.03, 0.26)	0.00
(-0.14, 0.15)	-0.03
(-0.18, 0.12)	0.04
(-0.12, 0.19)	-0.00
(-0.15, 0.15)	-0.02
(-0.09, 0.05)	0.19
(-0.43, 0.81)	
Not Hispanic/Latine	Ref	Ref	Ref	Ref	Ref	Ref	Ref	
Gender								
Man	-0.14
(-0.19, -0.08)	0.01
(-0.05, 0.07)	0.05
(-0.00, 0.11)	-0.05
(-0.11, 0.01)	-0.00
(-0.06, 0.06)	-0.00
(-0.03, 0.03)	-0.10
(-0.34, 0.14)	
								
Woman	Ref	Ref	Ref	Ref	Ref	Ref	Ref	
Race								
Black	-0.12
(-0.28, 0.05)	-0.00
(-0.21, 0.21)	0.02
(-0.18, 0.23)	-0.04
(-0.25, 0.18)	-0.15
(-0.37, 0.07)	-0.07
(-0.23, 0.09)	0.80
(0.01, 1.58)	
								
American Indian	0.14
(-0.13, 0.41)	0.01
(-0.33, 0.36)	0.03
(-0.29, 0.36)	-0.08
(-0.38, 0.21)	-0.02
(-0.34, 0.31)	0.07
(0.03, 0.11)	0.56
(-0.72, 1.83)	
								
Asian	0.02
(-0.10, 0.14)	-0.13
(-0.26, -0.01)	-0.06
(-0.20, 0.07)	-0.12
(-0.25, -0.00)	-0.15
(-0.28, -0.01)	-0.05
(-0.13, 0.03)	-0.53
(-1.06, -0.00)	
								
Not White, Black, Indian, or Asian	-0.07
(-0.20, 0.06)	0.06
(-0.08, 0.20)	0.02
(-0.13, 0.16)	0.00
(-0.15, 0.15)	-0.01
(-0.16, 0.14)	-0.01
(-0.08, 0.06)	0.01
(-0.62, 0.64)	
White	Ref	Ref	Ref	Ref	Ref	Ref	Ref	
Bold = P<0.05
