# Supplementary material for: Are people aware of the link between alcohol and different types of Cancer?
Source: BMC Public Health. 2021 Apr 15;21:734. doi: 10.1186/s12889-021-10780-2 (PMC8051079; doi:10.1186/s12889-021-10780-2)
Supplement: Supplementary file 3 — Additional file 3. Survey Questionnaire. The questionnaire developed by research staff. Questions were adapted from the Behavioral Risk Factor Surveillance System and prior studies of alcohol-cancer awareness. Citations for these are included in the manuscript text. [file 12889_2021_10780_MOESM3_ESM.docx]

Thank you for agreeing to take part in this survey about cancer risk factors.  This survey will take approximately 5 minutes to complete. Please click ‘Next’ to begin.

1. Do you think any of the following behaviors/factors could increase someone’s risk of cancer? (answer options: yes/no/don’t know)
   1. Smoking cigarettes
   2. Stress
   3. Drinking alcohol
   4. Exposure to sunlight/ultraviolet radiation
   5. Eating genetically modified foods
   6. Obesity
   7. Drinking coffee
2. Do you think your risk of developing the following types of cancer is increased by drinking alcohol? (answer options: yes/no/don’t know)
   1. Stomach
   2. Ovarian
   3. Breast
   4. Mouth & throat
   5. Brain
   6. Colon & rectal
   7. Liver
   8. Bladder
3. Have you ever consumed any alcoholic beverages in your lifetime? (answer options: yes/no)
4. During the past 30 days, **how many days** did you **have at least one drink** of any alcoholic beverage such as beer, wine, a malt beverage or liquor? (drop down – 0 to 30)
5. One drink is equivalent to a 12-ounce beer, a 5-ounce glass of wine, or a drink with one shot of liquor.

During the past 30 days, on the days when you drank, about **how many drinks PER DAY** did you drink on the average? (A 40 ounce beer would count as 3 drinks, or a cocktail drink with 2 shots would count as 2 drinks.) (drop down – 0 to 76)

1. Considering all types of alcoholic beverages, how many times during the past 30 days did you have 5 or more drinks (for men) or 4 or more drinks (for women) on an occasion? (drop down – 0 to 76)
2. Do you currently use a tobacco product (cigarettes, chewing tobacco, snuff, snus) every day, some days, or not at all? (answer options: every day/some days/not at all/don’t know)
3. If you knew that alcohol was a risk factor for cancer, would you:
   1. Seek more information online (answer options: yes/no)
   2. Seek more information from a healthcare provider (answer options: yes/no)
   3. Seek more information from a family member or friend (answer options: yes/no)
   4. Inform a family member or friend (answer options: yes/no)
   5. Seek out cancer screening (answer options: yes/no)
   6. Drink less alcohol (answer options: yes/no)
4. Have you ever been or are you currently diagnosed with cancer? (answer options: yes/no/prefer not to answer)
5. That you know of, has anyone in your family been diagnosed with cancer? (answer options: yes/no/don’t know/prefer not to answer)
6. Please describe your highest level of education (answer options: less than 9^th^ grade/9^th^ to 12^th^ grade, no diploma/high school graduate or GED/some college, no degree/associate’s degree/bachelor’s degree/graduate or professional degree)
7. Please describe your ethnicity (answer options: hispanic or latino/not hispanic or latino)
8. Please describe your race (answer options: white or caucasian/black or african american/american indian or alaskan native/asian/native hawaiin or pacific islander/mixed race/other)
9. What is your current age in years? (fill in the blank)
10. What is your gender? (answer options: female/male/non-binary or third gender/prefer to self-describe/prefer not to say)
11. What is your ZIP code? (fill in the blank)
